# Effectiveness of Culinary Nutrition Workshops on the Mood and Nutritional Interest of Inpatients With Mental Disorder

**DOI:** 10.7759/cureus.64691

**Published:** 2024-07-16

**Authors:** Cristina Vaqué-Crusellas, Blanca Ribot, Antoni Corominas-Díaz, Gemma Prat-Vigué, Anna Vila-Martí, Míriam Torres-Moreno, Montserrat Serra-Millàs, Quintí Foguet-Boreu

**Affiliations:** 1 Department of Social Sciences and Community Health, Faculty of Health Sciences and Welfare, University of Vic - Central University of Catalonia (UVic-UCC), Vic, ESP; 2 Research Group on Methodology, Methods, Models and Outcomes of Health and Social Sciences (M3O), Institute for Research and Innovation in Life Sciences and Health in Central Catalonia (IRIS-CC), Vic, ESP; 3 Department of Applied Health Sciences, Faculty of Health Sciences and Welfare, University of Vic - Central University of Catalonia (UVic-UCC), Vic, ESP; 4 Department of Psychiatry, University of Vic - Central University of Catalonia (UVic-UCC), Vic, ESP; 5 Department of Psychiatry, Althaia, Xarxa Assistencial Universitària de Manresa, Manresa, ESP; 6 Department of Experimental and Methodological Sciences, Faculty of Health Sciences and Welfare, University of Vic - Central University of Catalonia (UVic-UCC), Vic, ESP; 7 Department of Psychiatry, Vic University Hospital, Vic, ESP; 8 Department of Psychiatry, Faculty of Health Sciences and Welfare, University of Vic - Central University of Catalonia (UVic-UCC), Vic, ESP

**Keywords:** cooking, culinary, nutrition education, nutrition, culinary nutrition workshops, mood, interest in nutrition, mental disorder, mental health, inpatient psychiatry

## Abstract

Background

Promoting healthy eating habits through nutrition education programs is crucial to improving the overall health of people with mental disorders. This study aims to assess the effectiveness of culinary nutrition workshops on the mood and nutritional interest of hospitalized adults with mental disorders (MD) from the acute psychiatric unit of two general hospitals in Catalonia, Spain.

Methods

A pilot randomized control trial (RCT) was conducted with MD inpatient. Participants were randomly assigned to two groups: the intervention group received weekly culinary nutrition workshops with flexible participation and the control group continued routinary care. The interest in nutrition was analysed with an ad hoc item pre and post-intervention period. Mood changes were studied with a visual analog scale and analysed pre- and post-intervention periods as well as before and after every session. An ad hoc questionnaire was also used to assess the satisfaction of participants with the intervention. The obtained data were analysed at both descriptive and inferential levels.

Results

We included 81 participants, with a mean age of 45.3 (SD: 17.0); 66.7% were women, with 41 assigned to the intervention group and 40 to the control group. At the end of every culinary nutrition workshop, a statistically significant improvement in mood was observed in the intervention group (5.9 vs. 7.4 points, p<0.001). However, there were no significant differences in mood changes between the control and intervention groups after the intervention period (control group: 1.0 vs. intervention group: 1.5, p=0.473), while the nutritional interest was significantly improved after the intervention period intergroups (control group: 4.1 vs. intervention group: 37.2, p<0.001). The intervention was excellently valued by the participants regarding content, space, and health professionals, and generated interest and motivation, with scores above 9 on all these items.

Conclusion

The improvement of interest in nutrition and the satisfaction of hospitalised people with MD with the nutrition culinary workshops emphasize the need to design more comprehensive RCTs in hospitals and rehabilitation centers.

## Introduction

The prevalence of mental disorders (MD) has increased over time, and they are currently recognized as the major contributors to the overall burden of diseases [1]. In Spain, around 37% of the population suffers from some MD [2]. Specifically in Barcelona, 31.6% of women and 21.6% of men struggle with poor mental health perception [3]. This incidence has been increasing since the COVID-19 pandemic, which has highlighted the importance of good care for mental diseases [4].

MD can severely impact various aspects of a person’s life including their autonomy and functionality, time management and social and community integration [5]. Consequently, compared to those without mental health issues, individuals with MD typically report a lower level of life satisfaction [6], and have a higher average mortality rate than the general population with up to 25-30 years lower life expectancy [7]. The causes of increased mortality are multifactorial [8]; one of the primary contributing factors is the increased incidence of cardiovascular diseases (CVD) and their risk factors (smoking, obesity, metabolic syndrome, hypertension and diabetes) [7-8].

The high prevalence of CVD risk factors in people with MD, apart from being associated with first- and second-generation antipsychotic medication, which induces appetite and cravings for sweet foods and drinks and causes several metabolic abnormalities such as obesity, type 2 diabetes, hyperglycemia and dyslipidemia, is also linked with poor lifestyle choices [9]. People with MD are often less physically active [10] and have higher rates of smoking and drug use [11]. They also tend to follow diets of low nutritional quality lacking in fruits, vegetables, and fiber, and an excess of calories derived from processed foods high in sugar, salt and saturated fats, which deviate from a healthy diet [12]. Furthermore, some negative eating patterns such as fast-eating syndrome and disordered eating patterns which refer to poor eating practices that may not fulfill the criteria for eating disorders (meal skipping, food-related anxiety, feeling guilt associated with eating, loss of control around food or food restrictions), binge eating, food cravings, food addictions and night eating are also relevant issues in people with MD [12].

Recent studies have found a direct association between the habitual intake of ultra-processed foods and the appearance of symptoms of anxiety and mental disorders, as well as an increase in the appearance of depression [13]. The psychiatric symptoms themselves such as cognitive impairment and reduced executive function along with the side effects of the medication combined with a lower socioeconomic status and lack of social support make it challenging for individuals with MD to maintain a healthy diet [12].

However, dietary interventions promoting Mediterranean-style dietary patterns which consist in the consumption of whole, minimally processed plant foods including vegetables, fruits, cereals, legumes/pulses and nuts, extra virgin olive oil as the main culinary fat, moderate intakes of fish, eggs, poultry, dairy products and low consumption of red meat and sweets, have been effective in improving the dietary behaviors associated with CVD risk in individuals with MD [14]. Evidence supports that dietary intervention can also improve the knowledge and skills related to the planning and preparation of healthy meals [8].

These dietary changes positively influence the improvement of CVD risk factors by reducing high blood pressure and improving the glycemic and lipid profile of people with MD [8]. However, a meta-analysis found that their effect on body weight is small to moderate [12]. Anyway, they are positively associated with enhancements in mood, which is a long-lasting psychological arousal state with interacting dimensions related to energy, tension and pleasure [15]. Even though the evidence is limited, some studies demonstrated that dietary interventions are effective in improving the mood of individuals with MD by increasing their vitality, alertness, contentment, and well-being [16]. Both the behavioral changes and the improvements in dietary patterns are more significant in the programs that incorporate practical skills related to the planning, shopping, and preparation of healthy meals [8]. Practical and dynamic dietary interventions, in addition to raising awareness about the importance of following a healthy diet in people with MD, also enable them to gain skills and confidence in order to manage independently their food-related tasks [17]. People also feel emotional stability while cooking [18]. Although the effectiveness of dietary interventions evidences their timely implementation following the initial onset of the illness, most of the time dietetic nutrition care is not included in the interdisciplinary team of mental health services at the hospital level. For first episodes and serious decompensations, hospital admission is required, which is usually lengthy, with an average of 34.2 days [19]. During these extended hospitalization periods, various health promotion activities are offered as therapeutic measures adapting the Person-Centered Care (PCC) approach, which refers to care in which the individual’s values and preferences are elicited and once expressed, guide all aspects of their health care, supporting their realistic health and life goals [20]. However, healthy eating activities are rarely included in these initiatives. We consider that promoting a healthy diet, through participation in culinary nutrition workshops during the hospital stay, could improve mood and the interest to follow a healthy diet in people with MD. This paper would like to study the effectiveness of culinary nutrition workshops on the mood and nutritional interest of adults with MD hospitalized in the acute psychiatric unit in two general hospitals.

## Materials and methods

This pilot randomized control trial (RCT) was approved by the Ethics Committee of collaborating hospitals (Vic University Hospital (VUH) and Manresa University Hospital - Althaia (MUH); reference PR337-2022 and 22/75-2022, respectively). The VUH's psychiatry service is the referral center for inpatient mental health admissions for 160,646 inhabitants and Althaia serves 268,655 inhabitants (according to the 2019 census).

The research sample included all the adults with mental disorders admitted to these hospitals due to clinical decompensation that involved full hospitalization using a non-probability sampling. The inclusion criteria were: a) adult with mental disorders in acute or sub-acute in-patient regimes; b) medical consent to participate; c) willingness to participate in the study; d) capacity to understand the activities. Exclusion criteria were: a) having a score below 30 points on the global activity assessment scale [21]; b) suffering from an eating disorder measured by the Sick, Control, One, Fat, Food questionnaire (SCOFF) [22]; c) having any food restrictions or food allergy indicated in the clinical history.

The fieldwork was carried out during six months from October 2022 to March 2023, three months in each hospital. The patients were informed about the aim and implications of the study, and the ethical aspects related to their participation. A total of 81 inpatients, 27 men (33.3%) and 54 women (66.7%), became part of the study after signing the written informed consent. The primary clinical mental diagnoses were affective disorders (55.6%), followed by schizophrenia spectrum and other psychotic disorders (38.3%), and other disorders (6.2%).

**Figure 1:**
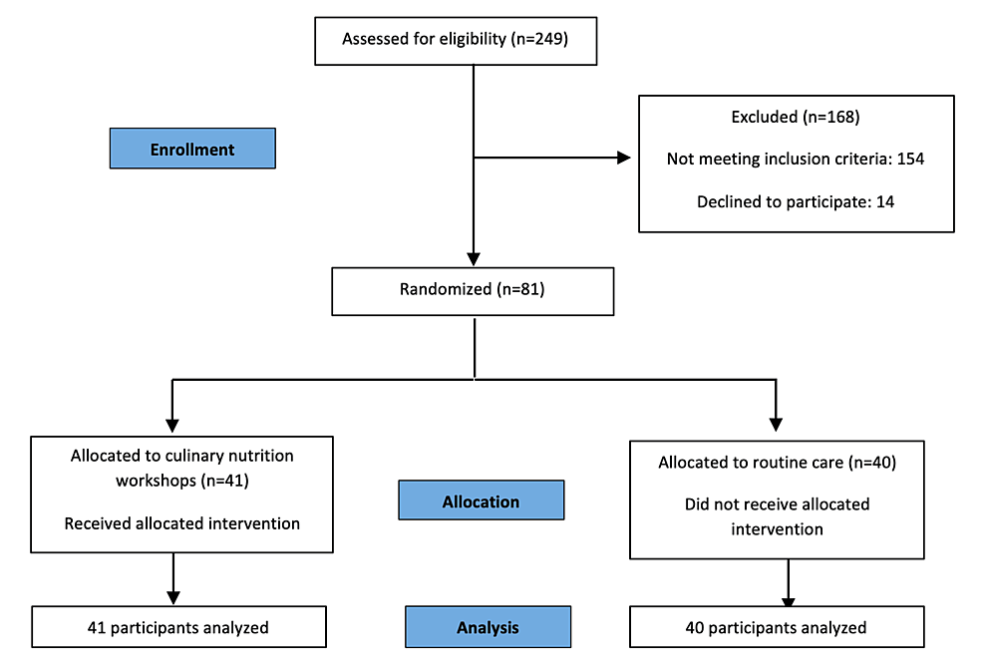
Flowchart of participants n = number of participants

After the baseline data collection, participants were assigned into two main groups (intervention and control groups) by simple random allocation in a 1:1 ratio. The method of blinding of this study was single blinding. The flowchart of CONSORT is shown in Figure 1.

Variables and instruments

Before starting the intervention, some variables were measured: sociodemographic data such as gender (male, female) and age (years); anthropometric data such as weight (kg) and height (cm) to calculate Body Mass Index (Kg/m^2^) (<18.5 underweight, 18.5-24.9 healthy weight, 25.0-29.9 overweight, and 30.0 and above obesity) [23]; and some clinical data as mental health diagnosis using Diagnostic and Statistical Manual of Mental Disorders, Fifth Edition (DSM-5) [24], first mental health episode (yes/no), length of stay (days), and tobacco habit (yes/no). Mental disorders were grouped into three groups: affective disorders, schizophrenia spectrum and other psychotic disorders, and others.

Other variables of the study were: eating behavior, which was collected only at the baseline, and mood, interest in nutrition and health perception which were collected before and after the intervention period. Furthermore, mood and health perception were also collected before and after each culinary nutritional workshop.

Eating behavior was studied by The Three-Factor Eating Questionnaire-21 (TFEQ-R21) which measures cognitive, behavioral, and emotional aspects of human eating attitudes [25]. TFEQ-R21 presents robust psychometric properties and asks participants to respond to 21 questions on a four-point Likert scale (where 4 means completely true, and 1 completely false) for items 1-20, and one on an eight-point numerical rating scale for item 21. Before calculating domain scores, items 1-16 were reverse coded, and item 21 was recoded as follows: 1-2 scores as 1; 3-4 as 2; 5-6 as 3; 7-8 as 4. Domain scores were then calculated as a mean of all items within each domain; hence, domain scores also ranged from 1 to 4. One domain is Cognitive Restraint (CR), and it refers to the conscious restriction of food intake to control body weight or to promote weight loss (six items). Uncontrolled Eating (UE) is another domain to measure the tendency to eat more than usual because of a loss of control over intake (nine items). The last domain is Emotional Eating (EE) expressing overeating during dysphoric mood states (six items). Higher scores are indicative of greater domains.

Health perception was studied using a visual analog health perception scale EuroQol-5D [26] with a range of 0 to 100 points where 0 represents the worst health perception at the moment, and 100 the best health perception. The individual marks the point on the line that best reflects the assessment of their overall health status at this moment.

Mood assessment was evaluated using a visual analog scale of mood from 0 to 10, where 0 corresponds to “I couldn’t be in a worse mood, right now”, and 10 to “I couldn’t feel in a better mood, right now”. Visual analog scale is simple to complete, ensuring a high rate of compliance, and it has been shown to possess high reliability and validity [27].

The interest in nutrition was studied by an ad hoc item asking the following: “To what extent are you interested in eating healthy to improve your health?”. Participants answered with a Likert scale from 0 to 100 points where 0 meant “not at all” and 100 meant “a lot”.

Lastly, four ad hoc items measuring the relevance of the content covered in the workshops, the motivation and interest they created, the competence of the experts leading them, and the suitability of the venue were used to assess participants' satisfaction with the nutritional culinary workshops. We used a Likert scale from 0 to 10, where 0 was “not at all”, and 10 was “a lot”.

Intervention

The intervention group received the possibility of attending nine weekly culinary nutrition workshops (Table 1) conducted by a nutritionist at their hospitals. The attendance at each culinary nutrition workshop was voluntary according to the Person‐Centered Care (PCC) model and based on participants' clinical progress. The workshops took place in VUH from October to January and in MUH from December to March. They were conducted in small groups (4-6 people) and each session lasted 50 minutes.

**Table 1:** Workshop topics, number of workshops, and attendees to the culinary workshops

Workshops	Aim of the workshop	Number of workshops conducted per topic	Participants average per workshop	Number of participants per topic
1- My plate	Build healthy eating habits using “My Plate” resource.	4	4.3	17
2- Fruits	Learn local fruits temporary nutritional value, and practice several ways to eat daily as a healthy snack, breakfast, dessert, or dish garnish.	3	4.7	14
3- Nutritional labelling	Raise awareness about nutrition facts labels and explain how to use it as a tool for maintaining healthy dietary practices.	3	4.7	14
4- Alternative meat protein	Review their meat intake and learn other ways to eat protein from plant-based alternatives cooking several recipes.	3	4.3	13
5- Vegetables	Learn local vegetables temporary nutritional value, and practice different ways to eat daily as cooked or raw vegetables.	3	4.7	13
6- Healthy snacks	Review the type of snacks consumed and learn how to prepare an easy, quick and healthy snack.	3	4.3	13
7- Healthy breakfast	Reinforce daily meal organization. Create healthy breakfast options without ultra-processed food.	3	4.3	13
8- Healthy beverages	Prioritize water as the most important beverage daily. Check the amount of sugar in fruit juice, sugar-sweetened beverages, energy beverages, infusions, vegetal drinks, … and elaborate healthy beverages.	3	4.0	12
9- Foods in a cereal group	Learn types of foods in cereal group, nutritional value, recommended portion, and practice different ways to eat daily.	2	5.0	10

Every workshop began with an explanation of its objectives and a brief overview of the dynamics (5-10 minutes), followed by participants cooking, eating, and discussing the nutritional information and health recommendations with the nutritionist. Meals prepared during the nutritional workshops were conceived to be simple, affordable, tasty meals based on Mediterranean diet recommendations. Participants were encouraged to attend as many workshops as possible during their hospital stay. We did not establish a minimum number of workshops to attend to be included in the intervention.

Statistical analysis

Data analysis was performed on two levels: descriptive statistics and inferential statistics. The results of descriptive statistics were expressed as mean (standard deviation) for continuous data and as n (%) for nominal data. Paired T-Test and Student t-test were used as appropriate to compare means, and the χ2-test to compare proportions.

Dummy variables were created according to the categorized diagnosis of the mental disorder, affective disorder being the category of reference. Multiple linear regression (MLR) models were conducted to explain the different variables of the study, adjusted for confounding variables.

MLR analysis was performed to explain the change in the mood after the intervention adjusted for the following covariates: intervention group (no, yes); gender of the participant (female, male); age (years); BMI (kg/m^2^); change in health perception; lengths of stay; first mental health diagnosis (no, yes); tobacco habit, dummy 1: affective vs. psychosis; dummy 2: affective vs. other diagnoses, and the number of nutritional workshops. Another MLR was performed to explain the change in the interest in nutrition after the intervention, adjusted for the intervention group (no, yes); gender of the participant (female, male); age (years); BMI (kg/m^2^); mood; eating behavior domains (cognitive restraint, emotional eating, and uncontrolled eating); tobacco habit, dummy 1: affective vs. psychosis; dummy 2: affective vs. other diagnoses, and number of nutritional workshops.

Statistical significance was set at P<0.05. All statistical analyses were performed using the SPSS statistical software version 29.0 (IBM Corp., Armonk, NY, USA) program.

## Results

The research involved 81 participants with an average age of 45.3 (17.0) years, and 66.7% of them were female.

The primary clinical mental diagnoses were affective disorders (55.6%), followed by schizophrenia spectrum and other psychotic disorders (38.3%), and other disorders (6.2%) (Table 2).

**Table 2:** Diagnoses of the participants

Diagnostic Criteria		n (%)
Affective disorders	Bipolar I Disorder	21 (25.9)
	Bipolar II Disorder	6 (7.4)
	Major Depressive Disorder	16 (19.8)
	Substance-Induced Disorders	2 (2.5)
Schizophrenia Spectrum and Other Psychotic Disorders	Schizophreniform Disorder	4 (4.9)
	Schizophrenia	8 (9.9)
	Schizoaffective Disorder	12 (14.8)
	Other Specified Schizophrenia Spectrum and Other Psychotic Disorder	7 (8.6)
Other disorders	Cluster B Personality Disorders	4 (4.9)
	Trauma and Stressor-Related Disorders	1 (1.2)

Table 3 shows the baseline characteristics of the participants, showing homogeneity across the two allocation groups.

**Table 3:** Baseline characteristics of participants for control and intervention group Values are expressed in † means (SD, standard deviation) or ‡ number (%). BMI: body mass index *P-value using two-sample t-test for continuous variables, λ2-test for categorical variables

		Control group, n=40	Intervention group, n=41	p-value^*^	Total, n=81
	GENERAL CHARACTERISTICS				
	Gender (women), %^‡^	28 (70.0)	26 (63.4)	0.530	54 (66.7)
	Age; years^†^	46.0 (16.9)	44.7 (17.3)	0.735	45.3 (17.0)
Mental clinical diagnosis:					
Affective disorders %^‡^		21 (52.5)	24 (58.5)	0.712	45 (55.6)
Schizophrenia spectrum and other psychotic disorders, %^‡^		17 (42.5)	14 (34.1)		31 (38.3)
Other disorders %^‡^		2 (50.0)	3 (7.3)		5 (6.2)
	BMI pre-intervention; Kg/m^2 †^	27.5 (6.0)	26.9 (5.8)	0.648	27.2 (5.8)
	<18.5 Kg/m^2^, %^‡^	1 (2.5)	2 (5)	0.776	3 (3.7)
	18.5-24.9 Kg/m^2^, %^‡^	16 (40.0)	16 (39.0)		32 (39.5)
	25-29.9 Kg/m^2^, %^‡^	10 (25.0)	13 (31.7)		23 (28.4)
	≥30 Kg/m^2^, %^‡^	13 (32.5)	10 (24.4)		23 (28.4)
	Smoker; %^‡^	17 (42.5)	11 (26.8)	0.138	28 (33.3)
	First mental health diagnosis; %^‡^	5 (12.5)	9 (22.0)	0.261	14 (17.3)
	Length of hospital stay; days^†^	32.2 (24.3)	30.0 (20.8)	0.666	31.1 (22.5)
	Health perception pre-intervention ^†^	61.3 (23.2)	58.4 (23.3)	0.584	60.0 (23.1)
	Mood pre-intervention ^†^	5.9 (2.5)	5.4 (2.5)	0.299	5.7 (2.5)
	Interest in nutrition pre-intervention^†^	37.3 (20.9)	42.7 (24.3)	0.284	40 (22.6)
	EATING BEHAVIOR (TFEQ domains)				
	Emotional eating domain^†^	14.8 (5.4)	14.9 (5.3)	0.981	14.8 (5.3)
	Uncontrolled eating domain^†^	21.2 (5.7)	19.3 (5.0)	0.112	20.2 (4.0)
	Cognitive restraint domain^†^	12.7 (3.5)	12.0 (3.8)	0.461	12.4 (3.6)

In 17.3% of cases, it was the patient’s first MD episode. The participants’ average length of hospital stay was 31.1 days, while the average BMI was 27.2 kg/m^2^, and 33.3% of them used to smoke. The three groups of mental disorder diagnoses were homogeneous regarding age, gender, BMI and length of hospital stay (data not shown). At baseline, no statistically significant differences were observed between the control and the intervention group in terms of eating behavior, mood, and interest in nutrition.

In total, our program developed 27 culinary nutrition workshops. The mean attendance of the participants at the workshops was 2.9 (ranging from 1 to 7); 80.5% attended at least two workshops. The control group continued with routine care and did not participate in any culinary workshops.

We also compared the mood in the intervention group before and after every nutrition culinary workshop using paired T-Test. The results showed a statistically significant post-workshop improvement in mood: pre-workshop: 5.9 (SD 1.9); post-workshop: 7.4 (SD 1.5); p<0.001, regardless of the topic covered in the workshops (Table 4).

**Table 4:** Pre- and post-workshop mood average according to the topics covered in the sessions. N = number of participants; SD = standard deviation

		N	Pre-workshop	Post-workshop	p-value
			Mean (SD)	Mean (SD)	
Mood	Vegetables	13	6.7 (2.2)	7.7 (1.9)	0.003
	Farinaceous	10	6.5 (1.2)	8.0 (1.0)	0.005
	Healthy beverages	12	6.5 (2.1)	8.0 (1.6)	0.002
	Healthy breakfast	13	4.7 (1.8)	6.7 (1.7)	0.002
	Fruit	14	7.4 (1.8)	8.8 (1.2)	0.003
	My plate	17	5.6 (1.5)	7.7 (1.3)	<0.001
	Nutritional labeling	14	5.5 (1.5)	6.9 (1.4)	0.001
	Healthy snacks	13	5.3 (2.1)	6.7 (1.9)	0.002
	Alternative meat protein	13	5.8 (1.7)	7.8 (1.7)	0.002

Furthermore, for the comparison between the control and intervention groups, we conducted a paired T-test on mood and interest in nutrition. The results show that the intervention was effective in significantly improving the interest in nutrition of the participants (p<0.001). However, when comparing the control and intervention groups according to the variation in mood (before and after intervention) they were not significant (Table 5).

**Table 5:** Impact of the culinary nutrition workshops on the changes in mood and interest in nutrition. Note: Changes in mood and interest in nutrition indicated the differences in mean before and after the intervention.

	Control group			Intervention group			p-value
	N	Mean	SD	N	Mean	SD	
Changes in mood	40	1.0	3.0	41	1.5	2.7	0.473
Changes in interest in nutrition	40	4.1	5.9	41	37.2	18.5	<0.001

We extensively examined the impact of culinary nutrition workshops on mood and interest in nutrition.

In order to explain the change in the mood after the intervention period, an MLR analysis was performed and adjusted for confounding variables. In this model (R^2^_c.100_=43.8; F_69,11_=6.666; p=<0.001), an increase of 1 point in the health perception led to a significant enhancement in the mood of the participants (beta: 0.076 points, p=<0.001).

Regarding the change in the interest in nutrition after the intervention period, another MLR analysis was conducted. In this model (R^2^_c.100_=50.7; F_68,12_=7.849; p=<0.001), the participants from the intervention group had 33.2 points more interest in nutrition than participants from the control group.

The participants highly appreciated the intervention, expressing satisfaction with the following aspects: applicability of the content 9.2 (SD 0.9), adequacy of the space 9.3 (SD 1.0), professional who conducted the workshops 9.7 (SD 0.5) and interest and motivation generated during the intervention 9.2 (SD 1.0).

## Discussion

The present study found favorable results in terms of increased interest in nutrition after workshops performed during hospital stays in an acute psychiatric unit.

To the best of our knowledge, there are few studies in this field conducted on psychiatric inpatients and none include previous eating behavior. Previous eating behavior can affect interest in nutrition. Therefore, taking this confounding variable into account strengthens the results obtained.

Another point to highlight is that the two groups (control and intervention group) were homogeneous in relation to baseline characteristics allowing an appropriate evaluation of this RCT, in which the intervention group (n=41) was offered nine weekly culinary nutrition workshops while the control group (n=40) continued with routine care. The possibility of participating in workshops was based on participants' clinical progress, which allowed the implementation of the Person‐Centered Care (PCC) model [20]. Furthermore, participants in the intervention group had the freedom to choose whether to attend the workshops or even stay for the entire session or leave earlier, if necessary. Therefore, the intervention was tailored to their needs, respecting their autonomy and dignity in making decisions. The implementation of the PCC facilitated a free decision to attend several workshops.

Due to the MD and the side effects of medication, engaging hospitalized people with MD in various productive tasks is complex, as they usually spend their hospital stay without significant occupations. For this reason, initially, participants from our study also showed limited interest in nutrition, since they had other vital priorities. The increase in interest in nutrition observed, as the culinary workshops were conducted, is similar to that observed in other studies where they found that the cooking courses also effectively improved the dietary selection of participants as well as the culinary skills and health status [8,28]. In patients with MD, cooking courses can also improve their self-esteem since witnessing the result of what they cook increases their empowerment, ability, and confidence to do things on their own [29]. Furthermore, a pilot study conducted in Australia found that nutrition culinary workshops are effective in improving nutrition knowledge and reducing poor nutrition habits of individuals with severe mental disease, especially causing a decrease in the consumption of soft drinks, energy drinks, and takeaway meals [8]. As far as we are concerned, interest in nutrition is not as explored as nutritional knowledge in MD patients. Interest in nutrition is a previous step to be able to improve the nutritional knowledge which is an important aspect to strengthen.

Although evidence on the impact of culinary nutrition workshops on the mood of hospitalized patients with MD is scarce, studies carried out on cancer patients showed that these interventions are effective in improving the quality of life and expectations in the face of a complex pathological situation which can contribute to improving their mood [19]. In our study, the culinary nutrition workshops effectively improved the mood of the intervention group after each session, providing them with a brief but effective diversion from the hospital admission scenario and offering them an opportunity to socialize and cultivate interpersonal interactions with other hospitalized people and health professionals. The social well-being gained from nutrition culinary programs has also been previously documented in several studies conducted with outpatients with MD [30,31]. Due to the social benefits and enjoyment of the culinary experience, at the end of every workshop participant’s mood was significantly enhanced even in inpatients. However, the pre- and post-intervention difference in overall mood was not significant, because the improvement of mood is temporary subjected to the workshops. One of the reasons is that the mood of hospitalized people does not depend only on discrete moments and is influenced by a variety of elements such as psychiatric interventions, psychological assistance, pharmacological treatment, and social support. Furthermore, our intervention consisted of offering culinary sessions weekly rather than daily, which could be insufficient to change their regular routines [16]. In addition, the absence of nutritionists in the healthcare team also made the change difficult. It may be interesting to integrate nutritionists into the healthcare provided to people with MD and to design appropriate nutrition education interventions for them.

At the end of the study, participants were highly satisfied with the intervention, which may have been impacted by the study design, which included practical culinary nutrition workshops and allowed flexible participation options to the participants. Having adequate spaces to carry out dynamic cooking and food manipulation practices also comforted participants. Moreover, the designed intervention was the result of the demand and interest shown by people hospitalized with MD and the healthcare professionals from these units.

Although the study’s participants expressed high levels of satisfaction with the intervention, we detected some limitations, such as using a non-representative sample size and the fact that, according to the study design, the intervention was not compulsory for all patients. It should be noted that the participants were not permanently hospitalized. Some were discharged during the intervention period, while others joined in the middle. Therefore, the intention-to-treat analysis provides a more realistic view of potential outcomes in a setting that follows a person-centered care methodology. However, there were no statistical differences in mood or interest in nutrition when comparing participants attending different numbers of nutritional workshops. Furthermore, in the multiple linear regression (MLR) analysis conducted, the number of workshops attended was not significant for the variables studied. We consider that this design has the potential to result in a larger and more comprehensive RCT with an increased number of participants which might enhance the statistical analysis procedures employed in the study. Additionally, some clinical data on cardiovascular risk factors, pharmacological treatment, and activities carried out during admission could also be controlled as confounding variables. As the functional abilities of individuals with MD vary greatly, it would be appropriate to design an intervention based on the functional level rather than the classification of mental diseases.

## Conclusions

Culinary nutrition workshops increased the interest in nutrition after the intervention, and raised the mood measured after the workshops, although it is not maintained at the end of the intervention. The interest in nutrition is a fundamental aspect of generating awareness about the importance of healthy eating habits. The increase in participants' interest in nutritional aspects as well as their satisfaction with the program emphasizes the need to provide nutritional care to people with MD during their prolonged hospital stay. We consider that dietary advice should also be provided upon discharge apart from pharmacological recommendations and other indications. Additionally, nutritional educational programs should be expanded in rehabilitation centers and at the community level for people with MD.
